# Acute medical unit comprehensive geriatric assessment intervention study (AMIGOS)

**DOI:** 10.1186/1745-6215-12-200

**Published:** 2011-08-24

**Authors:** Judi Edmans, Simon Conroy, Rowan Harwood, Sarah Lewis, Rachel A Elliott, Philippa Logan, Lucy Bradshaw, Matthew Franklin, John Gladman

**Affiliations:** 1 Division of Rehabilitation & Ageing, University of Nottingham, Medical School, Queen's Medical Centre, Nottingham, NG7 2UH, UK; 2 University of Leicester School of Medicine, Room 540, Level 5, Clinical Sciences Building, P.O. Box 65, Leicester Royal Infirmary, Leicester, LE2 7LX, UK; 3 Department of Health Care of Older People, Nottingham University Hospitals NHS Trust, Queen's Medical Centre, Nottingham, NG7 2UH, UK; 4 Division of Epidemiology & Public Health, University of Nottingham, Clinical Sciences Building, Nottingham City Hospital, Nottingham, NG5 1PB, UK; 5 School of Pharmacy, University of Nottingham, Pharmacy Building, University Park, Nottingham, NG7 2RD, UK

## Abstract

**Background:**

Many older people presenting to Acute Medical Units (AMU) are discharged after only a short stay (< 72 hours), yet many re-present to hospital or die within 1 year. Comprehensive Geriatric Assessment may improve patient outcomes for this group.

**Method:**

**Participants**

Patients aged > 70 years and scoring positive on a risk screening tool ('Identification of Seniors At Risk') who are discharged within 72 hours of attending an AMU with a medical crisis, recruited prior to discharge. Sample size is 400. Carers of participants will also be recruited.

**Intervention**

Assessment on the AMU and further out-patient management by a specialist physician in geriatric medicine. Assessment and further management will follow the principles of Comprehensive Geriatric Assessment, providing advice and support to primary care services.

**Design**

Multi-centre, individual patient randomised controlled trial comparing intervention with usual care.

**Outcome measurement**

Follow up is by postal questionnaire 90 days after randomisation, and data will be entered into the study database by a researcher blind to allocation. The primary outcome is the number of days spent at home (for those admitted from home), or days spent in the same care home (if admitted from a care home). Secondary outcomes include mortality, institutionalisation, health and social care resource use, and scaled outcome measures, including quality of life, disability, mental well-being. Carer strain and well being will also be measured at 90 days.

**Analyses**

Comparisons of outcomes and costs, and a cost utility analysis between the intervention and control groups will be carried out.

**Trial Registration:**

ISRCTN: ISRCTN21800480

## Background

Early and rapid hospital triage of patients with medical crises is undertaken in Acute Medical Units (AMUs) in the UK. Ninety-eight per cent of UK NHS hospitals have AMUs [1]. Many patients in AMUs have a very short length of stay (< 1-2 days) [2]. At least 10% of all attendees will be frail older people, identified by the presence of one or more geriatric syndromes [3,4]. Frail older people who present with a crisis to an AMU but who are discharged rapidly have poor outcomes: 58% subsequently re-present to the AMU and 29% die over the 12 months from the index presentation [4]. Hospital facilities are expensive and a hospital stay is usually the most costly episode in the patient's experience of care. Managers and clinicians are therefore under pressure to reduce avoidable hospital admissions. Despite a multitude of efforts to reduce hospital attendance and admission, the numbers are increasing year on year [5].

There is a considerable body of evidence supporting the effectiveness of complex interventions for frail older people in general [6], particularly Comprehensive Geriatric Assessment (CGA) [6,7]. However, delivering CGA in the context of an AMU is challenging - time, bed pressures and lack of appropriate expertise are all barriers - and a recent review of the literature was unable to identify any trials of CGA being delivered in AMUs [8].

We have clinically piloted a version of CGA suitable for use in older people presenting to AMUs, "interface geriatrics", in which a geriatrician makes a clinical assessment of older patients being discharged from an AMU, and plans any necessary aftercare including further assessment at home and liaison with primary care, intermediate care and specialist community services. This study will evaluate interface geriatrics compared with current management, using a randomised controlled trial with economic analysis.

## Objectives

The objective of the study is to examine if the intervention increases the number of days spent at home in high risk older patients discharged from an acute medical unit, over current management, and whether it is cost effective.

## Method

**Figure 1:**
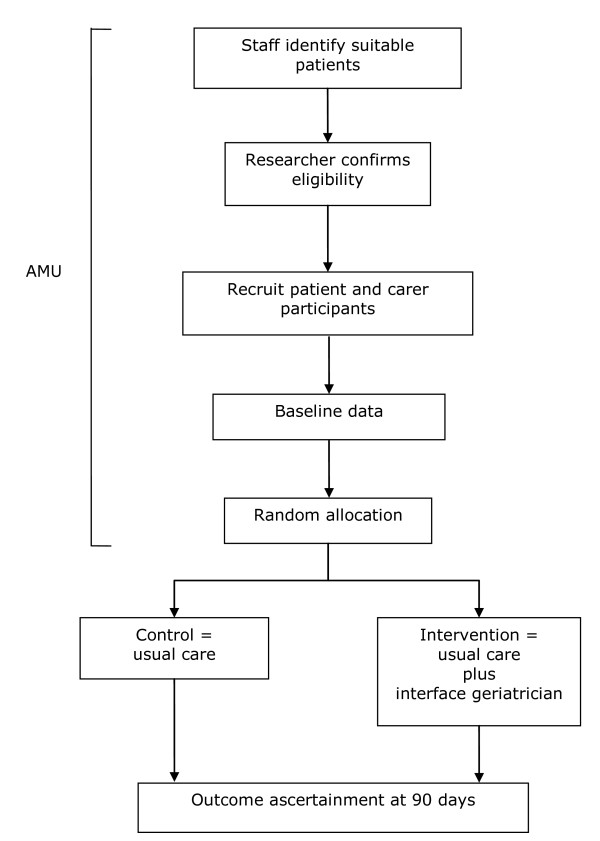
**Overall study outline of a comprehensive geriatric assessment intervention randomised controlled trial**. This illustrates the processes involved in a randomised controlled trial comparing comprehensive geriatrician assessment and intervention over current management for high risk older patients discharged from an acute medical unit.

A multicentre randomised controlled trial will be conducted. After informed consent and baseline data have been collected, participants will be randomised to usual care or the intervention arm, see Figure 1.

### Participants

**Figure 2:**
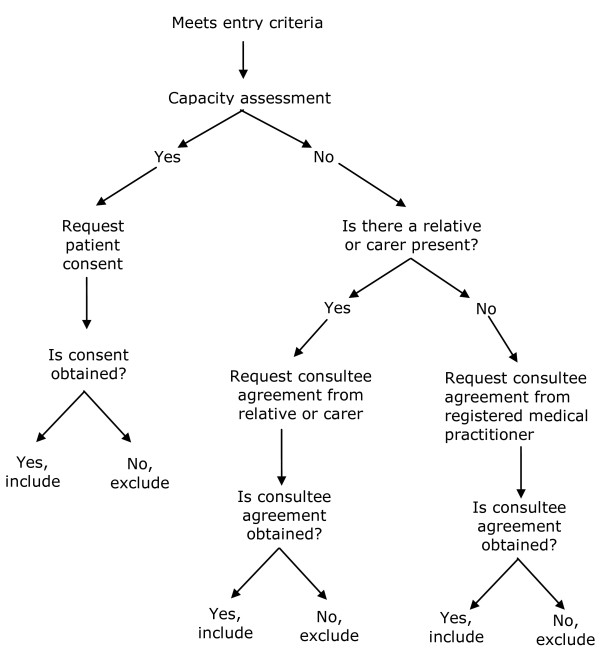
**Recruitment algorithm of a comprehensive geriatric assessment intervention randomised controlled trial**. This figure illustrates the recruitment algorithm to be used depending upon the presence or absence of capacity to consent to the study, and the presence or absence of a carer.

Patient participants will be patients who have been cared for on the AMU for less than 72 hours at the point of discharge, at either of two recruiting centres. Participants will be aged 70 years or over, and will be at high risk of future health problems as evidenced by a score ≥2/6 on the Identification of Seniors At Risk (ISAR) tool [9]. Patients excluded from the study will be: those not resident in the usual hospital catchment area; those without mental capacity to give informed consent and where there is no consultee available; those in whom an exceptional reason is cited by AMU staff why they should not be recruited (e.g. dangerous); and participants already participating in another intervention research project. Figure 2 shows the recruitment algorithm to be used depending upon the presence or absence of capacity to consent to the study, and the presence or absence of a carer.

Carer participants will be those identified as a carer of a patient participant by the patient participant or from the patient participant's clinical records.

### Baseline data

Baseline data for patient participants will comprise:

• Demographics: age, gender, ethnicity, marital status, residential status, education, social & financial questions

• Contacts and follow up information: usual and discharge (if different) address & telephone number, GP contact details

• ISAR score

• Health conditions: presenting problems, comorbidities and list of medications

• Cognitive function: Folstein MMSE [10]

• Personal ADL function: Barthel ADL Index [11]

• Health status: EuroQoL EQ5D [12] and ICECAP [13]

• Psychological well-being: GHQ-12 [14]

• Active receipt of palliative care services

• Resource use: health care (primary, secondary, intermediate and emergency services) and social services

Baseline data for carer participants will comprise:

• Carer strain: Caregiver Strain Index [15]

• Carer specific quality of life: CQLI-R [16]

• Health status: EuroQol EQ5D [12]

### Interventions

Standard care on the AMUs prior to recruitment for both the control and intervention groups will comprise an assessment by a consultant physician and attending medical team. Some patients will be assessed by members of a multidisciplinary team (physiotherapist, occupational therapist, nurse), as deemed appropriate by the AMU team.

Participants in the control group will receive no additional intervention over and above usual care after randomisation. Usual care is usually directed by the patient's general practitioner, but on some occasions will also include follow up and investigation by the acute medical team.

Participants in the intervention group will have an assessment by a geriatrician, who will also deliver or involve other agencies in the delivery of whatever aftercare they deem is necessary which may include: a review of diagnoses; a medication review; further assessment at home or in a clinic; advance care planning; and liaison with primary care, intermediate care and specialist community services. The intervention will commence immediately prior to discharge whilst on the AMU, continuing in the community and is largely expected to be complete within one month of randomisation.

### Clinical outcomes

The primary outcome will be "days at home". This will be calculated as 90 days from randomisation minus the number of days in this period each participant is in residential intermediate care units, in hospital (community hospital, psychiatric hospital or other acute hospital), in respite care or in a new care home placement or deceased [17]. This continuous outcome takes into account many different adverse outcomes including death and hospital re-admission or diversion to other settings as a result of health needs. This outcome will be ascertained from direct follow up of the participant, health and social service databases. Secondary outcomes will comprise:

• Death

• Institutionalisation

• Secondary care contacts (Emergency Department, AMU admissions, clinics)

• Personal Activities of Daily Living (Barthel ADL Index) [11]

• Self-reported falls over previous 90 days

• Psychological well-being (General Health Questionnaire, GHQ-12 [14])

• Health status (EQ5D [12] & ICECAP [18])

• Resource use and associated costs

• Carer strain: Caregiver Strain Index [15]

• Generic quality of life: EuroQol - EQ5D [12]

• Carer specific quality of life: CQLI-R [16]

Outcome measures will be collected by post, by phone or in person if necessary by a trained researcher not involved in the recruitment process and blind to allocation.

### Resource use

Costs will be estimated for each patient participant based upon health and social service resource use. Table 1 lists the resource use items that will be measured, how they will be determined, and what unit cost will be applied to them.

**Table 1:** Summary of resource use data to be collected

Parameters	Source	Unit costs
*Baseline primary care resource use per week*: home visit (community matron, specialist nurse, district nurse), attendance at day care centre, out-of-hours services	Clinical records via GP systems*	NHS and PSSRU reference costs
*Baseline personal social services per week: *home help, cleaner, private carer, community care assistant	Patient/carer participant report	
*Index intervention resource use*: length of stay and key interventions.	In situ data collection (clinical records or equivalent hospital systems)*	
*Follow up secondary care resource use*: clinic appointments and category of appointment; inpatient days and category of admission; A&E visits attendance at day hospital	In situ data collection (clinical records or equivalent hospital systems)*	
*Follow up use of emergency services* Paramedic and non-paramedic attended	Clinical records via ambulance service systems*	
*Follow up primary care resource use*: GP appointments; home visit (community matron, district nurse, specialist nurse), attendance at day care centre out-of-hours services admission to care home^	Clinical records via GP systems*	
*Follow up mental health resource use*: CPN visits Mental health admissions	Clinical records via mental health trust systems*	
*Follow up intermediate care resource use per week*: Home visits Admissions	Clinical records via intermediate care systems*	
*Personal social services:* home help, cleaner, private carer, community care assistant	Social services records and patient/carer participant report	

*Queries will be generated for the different data systems, to extract patient participant-based activity from baseline to the end of the follow-up period.

The cost of the interface geriatrician will be derived from a time log, kept by the participating geriatricians, of time spent on direct patient care activities (face to face contact and any other patient-related activities). Additional information on resource use will be collected on all participants in both arms (Table 1).

### Sample size

The primary outcome is the number of days spent at home in the 90 days after randomisation. Pilot data in a similar population demonstrated the mean number of days spent at home at 90 days was 63 with a standard deviation of 23 days. Using these data, a sample size of 200 in each group will have 90% power to detect a difference in the mean number of days at home of 7.5 days between the two groups (control group mean of 63 days and intervention group mean of 70.5 days) using a two-group t-test with a 5% significance level.

As days at home accounts for loss to follow up from events such as death, we will only need to slightly over-recruit to account for participants who withdraw completely for from the study (< 5% based on recruitment to date). Accordingly we aim to recruit up to 420 individuals overall.

### Randomisation

Participants will be randomly allocated using a 1:1 ratio to either the control group or to the intervention group. Randomisation will be stratified by centre.

### Sequence generation

A computer generated pseudo-random list using random permuted blocks of randomly varying size will be used. Access to the sequence will be confined to the Nottingham Clinical Trials Support Unit (CTSU) Data Manager.

### Implementation

The randomisation sequence will be created by the Nottingham CTSU http://ctu.nottingham.ac.uk/ctu in accordance with their standard operating procedures and held on a secure server. Investigators will access the treatment allocation for each subject by means of a remote, internet-based randomisation system developed and maintained by the Nottingham CTSU. The sequence of treatment allocations will be concealed from the researchers collecting the outcome data and the statistician until statistical analyses are complete.

### Blinding

It is not possible to blind participants to the intervention in this study, but outcomes will be collected by researchers who are blind to allocation and all analyses will be carried out blind to allocation.

### Statistical methods

#### Descriptive statistics

Continuous data that are approximately normally distributed will be summarised in terms of the mean, standard deviation; minimum, maximum and number of observations. Skewed data will be presented in terms of the median, lower and upper quartiles, minimum, maximum and number of observations. Categorical data will be summarised in terms of frequency counts and percentages. There will be no test of statistical differences or confidence intervals for differences between the intervention and control groups on any baseline variable, however analyses will be adjusted for prognostically important variables to improve the precision of the estimates of the intervention effect.

#### Univariate and multivariate analysis

The primary analysis will be based on the intention to treat principle, and will compare the primary outcome (days at home) between the two groups either using a two-sided t-test or a Mann-Whitney U test if the assumptions for the t-test are not satisfied, stratified by centre. We will use data from our recently completed cohort study (Acute Medicine Outcome Study) to explore the distribution of days at home in this population, constrained to take values between 0 and 90 days, to determine the most appropriate statistical model for the adjusted analysis. We will also use data from the cohort study to identify prognostically important variables for days at home in advance of the analysis of the randomised controlled trial. The intervention effect parameter will be presented as an estimate with 95% confidence interval.

We will also carry out a per-protocol efficacy analysis, looking at those patients who actually received the intervention.

All continuous secondary outcomes will be analysed using linear regression models, while logistic regression and Poisson regression models will be used for dichotomous and count data, respectively. Mortality will be analysed using Cox proportional hazards regression. All adjusted analyses will include centre in the model and other baseline variables used in these models will be specified in advance of the analysis based on the results of our prior cohort study. All statistical tests will be two-sided and performed using a 5% significance level with no adjustment for multiplicity.

We anticipate a greater recognition of the need for palliative care services in this population and will test for interaction between the intervention and terminal care status. If interaction is present, the results will presented as sub-group analyses. Other pre-specified sub-group analyses will be carried out according to the ISAR score, residency at baseline (care home vs. no care home) and cognition (MMSE ≤27), as these are prognostically important baseline indicators of adverse outcomes.

#### Economic analyses

Costs will be constructed from the perspective of the NHS and personal social services (PSS). 90 days of resource data will be collected for each patient participant. Costs will be estimated for each patient participant in the study. The costs will be calculated as resource use multiplied by the unit cost of the specific resource.

Health and social care resource use data will be valued using published unit cost data [19].

A cost-effectiveness analysis comparing specialist geriatric assessment, intervention and community-based follow-up following on from an acute admission to standard care will be carried out. Benefits to the patient participant will be measured with Quality-Adjusted Life Years (QALYs), which will be generated using EQ5D data, assuming homogeneity across treatment groups in any insensitivity of the EQ5D to our patient participants. The time horizon of the economic analysis will incorporate the lifetime of the cohort, through extrapolation of quality-adjusted survival from the end of the intervention period. These will be combined with cost data to generate incremental cost effectiveness ratios and Incremental Net Benefit (INB) statistics [20].

No discounting of intervention costs will be necessary as the intervention period is less than 12 months. QALYs will be discounted at 3.5%. Incremental cost-effectiveness analysis will be undertaken in the absence of convincing dominance by any treatment alternative. Probabilistic sensitivity analyses and cost-effectiveness acceptability curves will used to assess the risks posed by uncertainty in the estimated incremental net benefit statistics. Value of information and value of distributional information analyses will also be undertaken if appropriate.

#### Procedures for missing, unused and spurious data

If there is less than 5% missing data we will use the complete dataset but where there is more than 5% missing data our preferred method will be multiple imputation. We will use the multiple imputation procedures in Stata to impute missing values, including all baseline variables. We will then combine the results using Rubin's rules [21] for multiply imputed data.

Additionally we will assess the baseline characteristics of those who have dropped out or with large amounts of missing data in order to see if there is any differential loss to follow up which may result in bias.

Where extreme outliers are identified during model checking, sensitivity analyses will be carried out including and excluding the extreme outliers in order to test the robustness of the findings.

### Ethical approval

The protocol was given a favourable opinion by the Nottingham 1 Research Ethics Committee (reference 10/H0403/1).

## Abbreviations

ADL: Activities of Daily Living; AMIGOS: Acute Medical Unit Comprehensive Geriatric Assessment Intervention Study; AMU: Acute Medical Unit; CGA: Comprehensive Geriatric Assessment; CQLI-R: Caregiver Quality of Life Index-Revised; CTSU: Clinical Trials Support Unit; EQ5D: Euroqol 5-dimension quality of life scale; GHQ-12: General Health Questionnaire 12-item version; GP: General Practitioner (family doctor); INB: Incremental Net Benefit; ICECAP: ICEpop CAPability index; a measure of capability for the adult population; ISAR: Identification of Seniors At Risk; MMSE: Mini Mental State Examination; NHS: (United Kingdom) National Health Service; NIHR: National Institute for Health Research; PSS: Personal Social Services; QALYs: Quality Adjusted Life Years; UK: United Kingdom

## Competing interests

The authors declare that they have no competing interests.

## Authors' contributions

JE participated in the design of the study, and drafted the manuscript. SC conceived the study, participated in the design and drafted the manuscript. RH conceived the study, participated in the design and drafted the manuscript. SL conceived the study, participated in the design and drafted the manuscript. RE conceived the study, participated in the design and drafted the manuscript. PL participated in the design of the study. LB participated in the design of the study and drafted the manuscript. MF participated in the design of the study. JG conceived the study, participated in the design and drafting the manuscript. All authors read and approved the final manuscript.
